# Enhanced magnetic properties through tailoring of morphology of electrospun strontium hexaferrite nanofibers

**DOI:** 10.1038/s41598-025-04493-4

**Published:** 2025-07-02

**Authors:** Nishtha Vats, Saket Sanjay Phadkule, Shrutidhara Sarma, Devendra Singh Negi, Badri Vishal, Sampat Raj Vadera, Durgamadhab Mishra

**Affiliations:** 1 Department of Physics, Indian Institute of Technology, N H 62, Nagaur Road, Karwar, Jodhpur, 342037 Rajasthan India; 2 Department of Mechanical Engineering, Indian Institute of Technology, N H 62, Nagaur Road Karwar, Jodhpur, 342037 Rajasthan India; 3 Department of Metallurgy and Materials Engineering, Indian Institute of Technology, N H 62, Nagaur Road Karwar, Jodhpur, 342037 Rajasthan India; 4 KPV-LAB, KAUST Solar Center King Abdullah University of Science and Technology, Jeddah, KSA Saudi Arabia

**Keywords:** Morphology, Electrospinning, Nanofibers, Strontium hexaferrite, Permanent magnets, Polymer, Materials science, Physics

## Abstract

This work reports a simple yet unique way of tailoring the morphology of M-type Strontium Hexaferrite, SrFe_12_O_19_ (SFO) particles inside the nanofibers. The reported materials were synthesized by the polymer-sol-assisted electrospinning technique followed by calcination. Calcination temperature and rate of heating play significant role in controlling the morphology of SFO nanofibers in terms of shapes, size and distribution thereby having a direct bearing on the resultant magnetic properties. The synthesized materials were investigated using X-ray Diffraction (XRD), Field Emission Scanning Electron Microscopy (FESEM), High Resolution Transmission Electron Microscopy (HRTEM), Thermogravimetric-Differential Thermal Analysis (TG-DTA), and Magnetic Property Measurement System (MPMS). The XRD analysis shows formation of almost phase pure SFO with very minute quantities of secondary phase as hematite. The FESEM images show wide range of morphologies of SFO nanofibers and specifically the nanoparticles present within the nanofibers (from irregular to platelet-like shape) thereby demonstrating the tailoring of morphologies of not only of nanofibers but nanoparticles within the nanofibers simply through variations of two parameters viz. calcination temperature and rate of heating. The TG-DTA results show that complete decomposition of PVP and formation of the SFO crystallization takes place at a much lower temperature of 422 ^o^C. The room temperature hysteresis measurement showed that the sample calcined at 800 ^o^C at heating rate of 10 ^o^C/min has shown enhanced magnetic properties (M_s_, M_r_, and H_c_) than the samples calcined at 600 ^o^C and 950 ^o^C. The HRTEM studies show that the variation in heating rate also yields interesting variations in morphology of nanoparticles and their arrangement within nanofibers. This research work has resulted in a unique pathway of synthesizing nanofibers with controlled and variable morphology.

**Supplementary Information:**

The online version contains supplementary material available at 10.1038/s41598-025-04493-4.

## Introduction

Morphology, i.e., the shape, size, and structure, of materials plays a crucial role in determining their functional properties. Particularly, in case of magnetic materials, the variations in the morphology can show significant influence on its magnetic properties, especially on anisotropy thereby expanding the application domains in green and sustainable technology for the future^1^. Magnetic nanostructures and nanoparticles of different morphologies can lead to different kinds of anisotropies (surface or shape anisotropy) and novel spin textures, which are not only interesting for fundamental studies but also have importance for energy efficient devices^2^. An elongated nanoparticle or nanowire, for example, tends to exhibit a higher magnetic anisotropy along its long axis^3^. The high values of surface-to-volume ratio in nanomaterials also have significant impact on their magnetic properties. Hence, through controlled morphological changes, i.e., tailoring of shape, size, and structure in the form of nanofibers and nanoparticles, the magnetic properties of the materials can be enhanced, making them suitable for use in various applications including for permanent magnets.

Permanent magnets (PMs) find extensive applications in electric motors and generators, including those utilized in electric vehicles, hybrid vehicles, wind turbines and in a range of industrial applications. They play a vital role in enhancing the efficiency and overall performance of these systems, which are promising for green and sustainable future^4^. M-type Strontium Hexaferrites (SFOs) are highly promising materials for PM applications due to their high values of saturation magnetization, coercivity, Curie temperature, and magnetocrystalline anisotropy together with showing excellent chemical stability, mechanical hardness, strong corrosion resistance, and are quite cost-effective^5,6^. It is possible to tailor the magnetic properties of SFO by controlled variations in the morphology thereby further enhancing the scope of their applications.

Therefore, several studies have focused on the tailoring the morphology of materials adopting various synthesis methods and processing parameters showing their impact on magnetic properties. Eikeland et al. reported the formation of SrFe_12_O_19_ nanocrystallites of different morphologies by using conventional sol-gel (CSG) as well as modified sol-gel (MSG) synthesis routes^7^. In the MSG synthesis, the produced nanocrystals are strongly intergrown thereby resulting in enhanced magnetic properties as compared to CSG route. Lu et al. reported the synthesis of strontium ferrite micro- and nanofibers using sol-gel and electrospinning processes, respectively and their subsequent calcination above 750 ^o^C^8^. Nanofibers (diameter 100 nm) of SFO show higher value of magnetic coercivity 523.6 kAm^− 1^ as compared to 361.9 kAm^− 1^ for SFO microfibre (diameter 1 μm). The difference in coercivity between microfibers and nanofibers has been attributed to shape anisotropy energies. Jing et al. synthesized SFO nanoribbons by varying PVP concentration in the spinning solution wherein they reported variations in the ribbon-width, surface-flatness and particle-size^9^. They also reported that the M_s_, M_r_ and H_c_ values were found to increase with broadening of ribbon-width and the highest M_s_ and H_c_ values of 67.9 emu·g^− 1^ and 7.31 kOe, respectively were reported. Wang et al. synthesized SFO microtubules by sol-gel template method^10^. They reported that with increase in the calcination temperature, the tube walls became thicker and looser with external diameters between 8 and 13 mm; the wall thicknesses ranged between 1 mm and 2 mm. In this study they reported the highest H_c_, M_s_, and M_r_ as 7.12 kOe, 70.1 emu/g and 42.4 emu/g, respectively for the Fe^3+/^Sr^2+^ molar ratio of 11.5 and at calcination temperature of 850 ^o^C. L. Cong-Ju et al. synthesized strontium hexaferrite micro/nanostructures with different morphologies viz. microtubes and nanorods, using electrospun thermoplastic ester elastomer (TPEE) micro/nanofibers as the template^11^. The results indicate that prolonged annealing time has significant effect on their saturation magnetization (M_s_) and coercivity (H_c_) values. Advanced studies on electrospun SFO nanofibers using TEM and HRTEM have been conducted, where lattice fringe distances and the corresponding hkl planes of SFO were calculated^12,13^. These observations revealed that the SFO nanoparticles within the nanofibers possess a single crystalline structure.

The vast literature demonstrate that tailoring of morphology of these materials can be achieved either by varying the synthesis process and/or the process parameters as mentioned above. Amongst these techniques, the electrospinning is a simple, versatile, and cost-effective way to synthesize 1-D ultrafine fibers with diameters ranging from few µm down to tens of nm using a polymer melt or solution forced through a spinneret via high voltage DC source^5,6,14,15^. In this technique, one-by-one continuous nanofibers from various polymers can be mass produced^16^. The parameters such as polymer’s concentration, molecular weight, and the electrical conductivity of the solution play a significant role in controlling the formation of electrospun nanofibers^17,18^.

In this work, the as-spun nanofibers were synthesized through polymer-sol assisted electrospinning route. The as-spun nanofibers were subjected to calcination to obtain SFO nanofibers. The morphology of these SFO nanofibers has been uniquely controlled by systematic variation of the calcination parameters viz. heating rate and calcination temperature, which results into decomposition of the polymer and leading to interesting morphology of SFO nanoparticles arranged in nanofibers. Thus, a novel, yet simple process is developed to control the morphology at two levels, not only of nanofibers but also that of nanoparticles inside these nanofibers. The variation of the microstructures, crystallite size, nanofiber diameter, and magnetic properties of as-spun nanofibers and the SFO nanofibers, obtained after calcination, have been investigated by using different techniques viz. XRD, SEM, HRTEM, VSM and TG-DTA.

## Results and Discussion

### Structural characterization

**Fig. 1:**
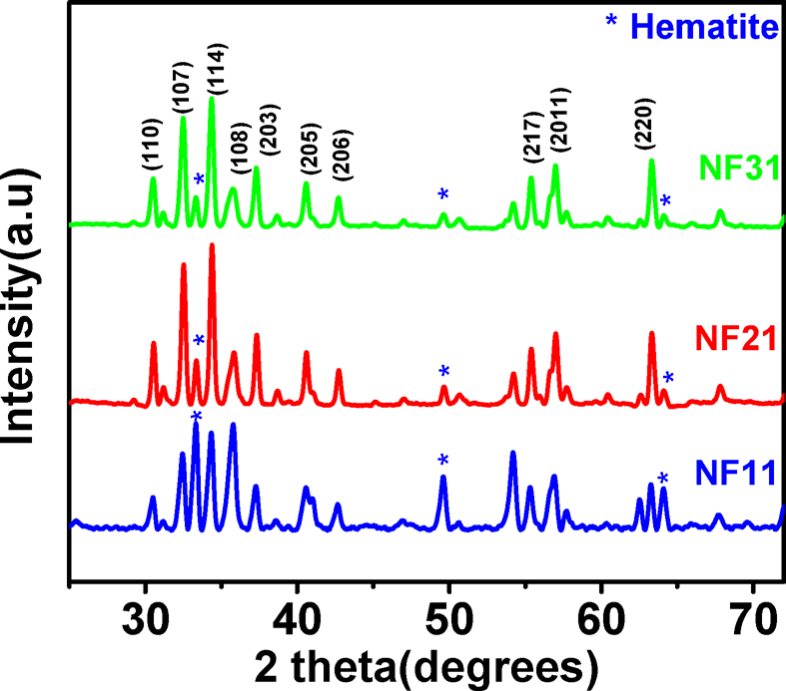
XRD patterns of SFO nanofibers calcined at 600 ^o^C (NF11), 800 ^o^C (NF21) and 950 ^o^C (NF31).

**Effect of calcination temperature.** Figure 1 depicts the XRD pattern of nanofibers NF11, NF21 and NF31 calcined at different temperatures with constant heating rate of 2 ^o^C/min. All the three samples show formation of hexaferrite phase with some impurities of hematite. Amongst these three samples, the nanofiber calcined at 600 ^o^C, NF11 has the highest intensity peak corresponding to hematite phase. In a typical solid state synthesis, the hexaferrite phase forms at temperatures exceeding 800 ^o^C, however, it is interesting to note that in this case the sample heated at 600 ^o^C shows formation of hexaferrite phase in the nanofiber. This may be attributed to generation of excess heat due to combustion of polymeric component of the electrospun fibers. Further, the intensity of the peak corresponding to hematite phase decreases significantly at higher calcination temperatures of 800 ^o^C and 950 ^o^C. Similar observation have been reported by other investigator as well^19^. It is also to be noted that complete removal of hematite could not happen most likely due to excess Fe used during synthesis process^20^. Moreover, the calcination temperature was not increased further inorder to stop nanoparticle agglomeration^19^.

For quantitative analysis of the XRD spectra, the lattice parameters (a, c), unit cell volume and c/a ratios for the hexagonal crystal system were calculated using the following equations.

1 $$\:\frac{1}{{d}^{2}}=\left(\frac{4}{3}\frac{{h}^{2}+hk+{k}^{2}}{{a}^{2}}+\frac{{l}^{2}}{{c}^{2}}\right)$$

2 $$\:2d\:\text{sin}\theta\:=n\lambda\:$$

3 $$\:V=\frac{{\sqrt{3}a}^{2}c}{2}$$

where d is interplanar spacing, θ is the diffraction angle, λ = 1.5406 Å is X-ray wavelength, n is order of the diffraction peak, h, k and l are Miller indices.

Using Scherrer formula^21^the crystallite size (D) was calculated

4 $$\:D=\frac{k\lambda\:}{\beta\:\text{cos}\theta\:}$$.

where k = 0.9 is a constant, and β is full-width at half-maximum of diffraction peak. All these structural parameters are shown in Table 1 for comparison.

**Fig. 2:**
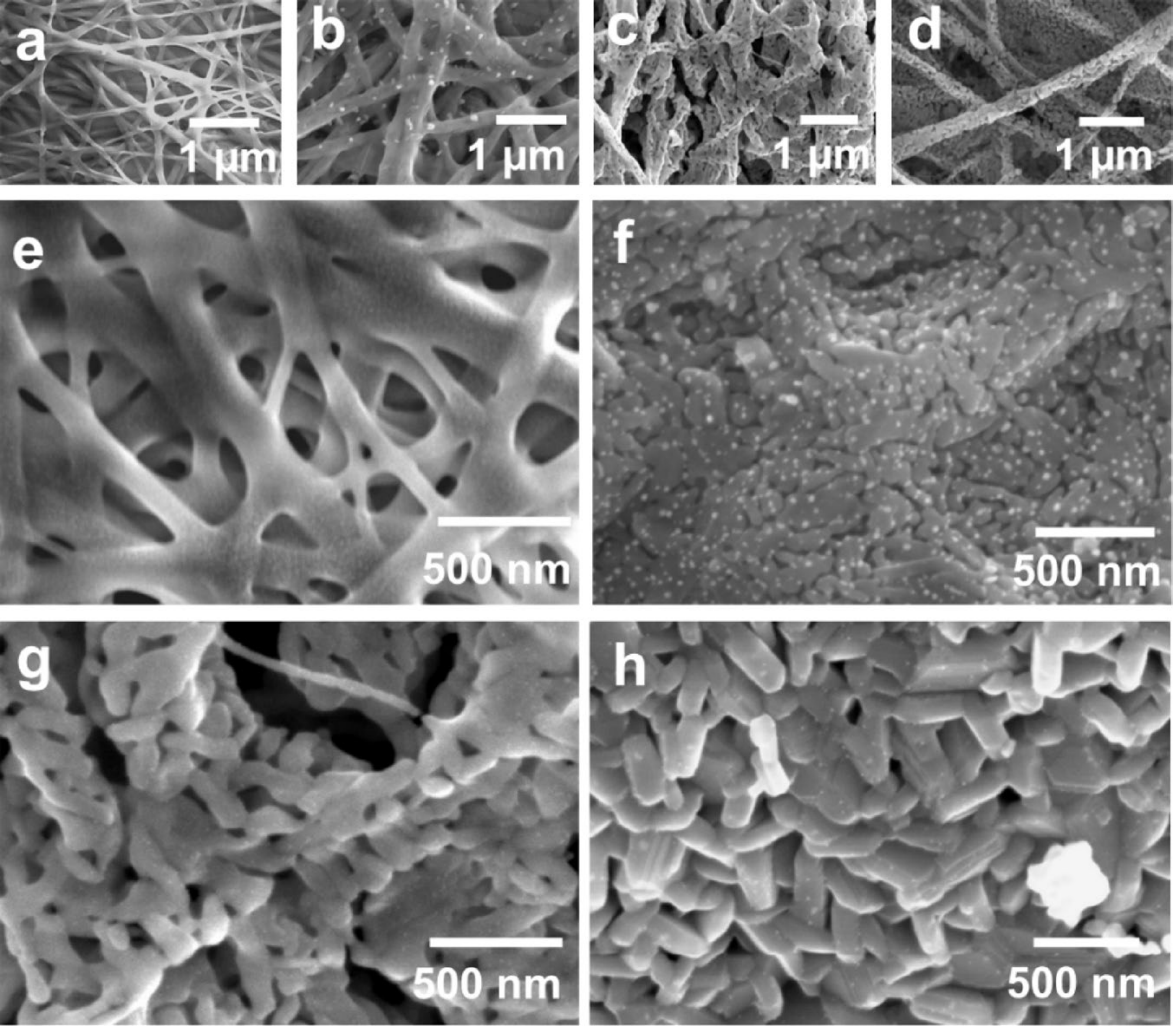
FESEM images of (**a**, **e**) as-spun nanofiber, (**b**–**d**) SFO nanofibers NF11, NF21, and NF31 at 80,000× magnification (**f**–**h**) SFO nanofibers NF11, NF21, and NF31 at magnifications of 120,000×.

The lattice constants (*a* and *c*) for the SFO samples are similar to those reported in the literature^22,23^. The unit cells parameters does not show any significant variation for all samples obtained after calcination at three different temperatures, except for NF31 which has a higher *a* value. The unit cell volume as calculated from the Eq. (3) indicates that the value approaches the bulk value of SFO with the increase in the calcination temperature. The observations also suggest that almost complete SFO crystallization occurs at 800 ^o^C. Further, the calculated crystallite size is found to increase with calcination temperature from 600 ^o^C to 800 ^o^C. However, the sample NF31 calcined at 950 ^o^C is found to have lower crystallite size compared to NF21 calcined at 800 ^o^C. This might be due to broadening of the peak due to strain within the crystals and at the interface of two particles as seen in the electron micrographs as shown in Fig. 2h.

Since heating rate plays an important role in controlling the morphology further investigations were carried out on as prepared nanofibers by heating them to 800 ^o^C at different heating rates of as described in the synthesis method section. The calcination temperature of 800 ^o^C was chosen for further studies since almost complete crystallization of SFO takes place at this temperature.

**Table 1:** Lattice constants (a, c, c/a ratio and V), crystallite size(D) of the SFO nanofibers NF11, NF21 and NF31.

Sample name	Lattice constants				Crystallite size
	a(Å)	c(Å)	c/a	V(Å^3^)	D(nm)
**NF11**	5.85 ± 0.002	22.96 ± 0.006	3.92	680.47	23.73
**NF21**	5.85 ± 0.002	22.87 ± 0.001	3.91	677.81	31.82
**NF31**	5.86 ± 0.002	22.93 ± 0.002	3.91	681.91	27.55

### Effect of heating rate

**Fig. 3:**
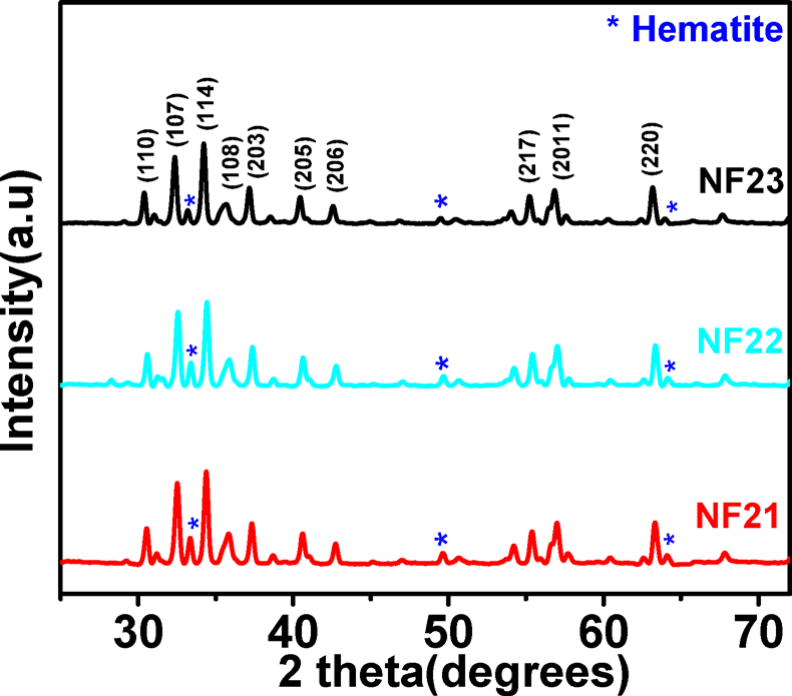
XRD patterns of SFO nanofibers calcined at 800 ^o^C at heating rates of 2 ^o^C/min (NF21), 6 ^o^C/min (NF22) and 10 ^o^C/min (NF23).

Figure 3 depicts the XRD spectra of SFO nanofibers NF21, NF22, and NF23 calcined at 800 ^o^C with heating rates of 2 ^o^C/min, 6 ^o^C/min, and 10 ^o^C/min, respectively. The diffraction peaks confirm the formation of hexagonal SFO (ICSD file no. 069023) phase. Impurity phase corresponding to hematite has also been observed in all the samples irrespective of the heating rate. The lattice parameters have been calculated for all the three samples by using Eq. 1. It is found that out of the three samples, the sample NF23 only, obtained by heating the nanofiber to 800 ^o^C at a heating rate of 10 ^o^C/min, shows lattice parameter values close to bulk SFO. The unit cell volume also increases with increasing heating rate with NF23 exhibiting close to the bulk value. All these structural parameters are shown in Table 2. The studies thus show that the heating rate affects the phase formation process. The higher heating rate might be assisting in better thermal decomposition of the polymer creating uniform temperature and decreasing the activation energy for crystallization thus leading to a unit cell closer to the bulk value of SFO^24^. Further, the crystallite size, as calculated from Scherrer’s equation, has been found to increase with increase in the rate of heating again suggesting that higher heating rate results in better growth of the crystallites.

**Table 2:** Lattice parameters (a, c), c/a ratio, volume of unit cell, crystallite size(D) of the SFO nanofibers NF21, NF22, and NF23.

Sample name	Lattice constants				Crystallite size
	a(Å)	c(Å)	c/a	V(Å^3^)	D( nm)
**NF21**	5.85 ± 0.002	22.87 ± 0.002	3.91	677.81	31.82
**NF22**	5.84 ± 0.002	22.80 ± 0.002	3.90	673.43	33.86
**NF23**	5.88 ± 0.002	23.02 ± 0.002	3.91	689.27	36.28

## Morphological Characterization

### Effect of calcination temperature

Figure 2 illustrates the SEM images of samples NF11, NF21 and NF31 calcined at different temperatures at magnifications of 80,000× and 120,000×, respectively. The as-spun nanofibers depicted in Fig. 2a exhibit a continuous network of interconnected and intercrossing structures extending in micrometer range along one direction and having a smooth surface with an average width of 246 ± 7 nm, which is amorphous in nature. As the calcination temperature increases the fiber structures are retained and nanoparticles of SFO phase are formed within the fiber structure (Fig. 2b-d). As the crystallization and formation of SFO takes place the surfaces appear to be rough due to the formation of irregular shapes within the fiber. The average fiber width decreases with increasing calcination temperature having values of 149 ± 11 nm, 138 ± 7 nm and 130 ± 5 nm for NF11, NF21 and NF31, respectively. This occurs due to decomposition of PVP polymer and shrinking of the polymer fiber diameter with increasing the temperature. The reduction in width is also associated with increased porosity in the fibers. The porosity is higher in NF21 and NF31 samples compared to NF11, which indicates that the polymer decomposition has not taken place at 600 ^o^C. Alibe et al. observed that for the decomposition of PVP, a minimum calcination temperature of 740 ^o^C was required in presence of other dispersed solutes or particles^25^. The porosity should be higher in NF31 compared to NF21 due to higher temperature. However, PVP decomposition allows grain growth at higher temperatures and create larger nanoparticles in contact with each other.

Figure 2e-h show higher magnification SEM images for as-prepared, NF11, NF21 and NF31 samples wherein one can clearly see the formation of nanoparticles and arrangement of these nanoparticles within the fibers. The average SFO particle sizes within the fiber in NF11, NF21 and NF31 are 39 ± 1 nm, 89 ± 4 nm, and 113 ± 4 nm, respectively. The retaining of SFO nanoparticles at temperature as high as 800 ^o^C may be attributed to high concentration of PVP molecules in as prepared nanofibers which could act like stabilizing as well as capping agent for nanoparticle growth^26^. It prevents agglomeration and suppresses grain growth. Therefore, NF11 sample has smaller particle size. However, as the decomposition of PVP starts, particle size increases as observed in NF21 and NF31 samples. Further, the diameters of as prepared nanofibers may also be constraining the growth of nanoparticles within the nanofibers despite heating the samples at high temperature. It is interesting to note that along with the size of the particles their morphology also changes within the fiber with increasing the calcination temperature. NF11 (Fig. 2f) has irregular shape with lamellar arrangement due to the partial decomposition of polymer. NF21 and NF31 (Fig. 2g, h) have anisotropic rod like shapes stacked next to each other. Moreover, in NF31 platelet morphologies can also be observed, which is clear evidence of formation of SFO phase. The width of the fibers and the particle morphologies are likely to have significant impact on the magnetic properties of the assembly.

### Effect of heating rate

**Fig. 4:**
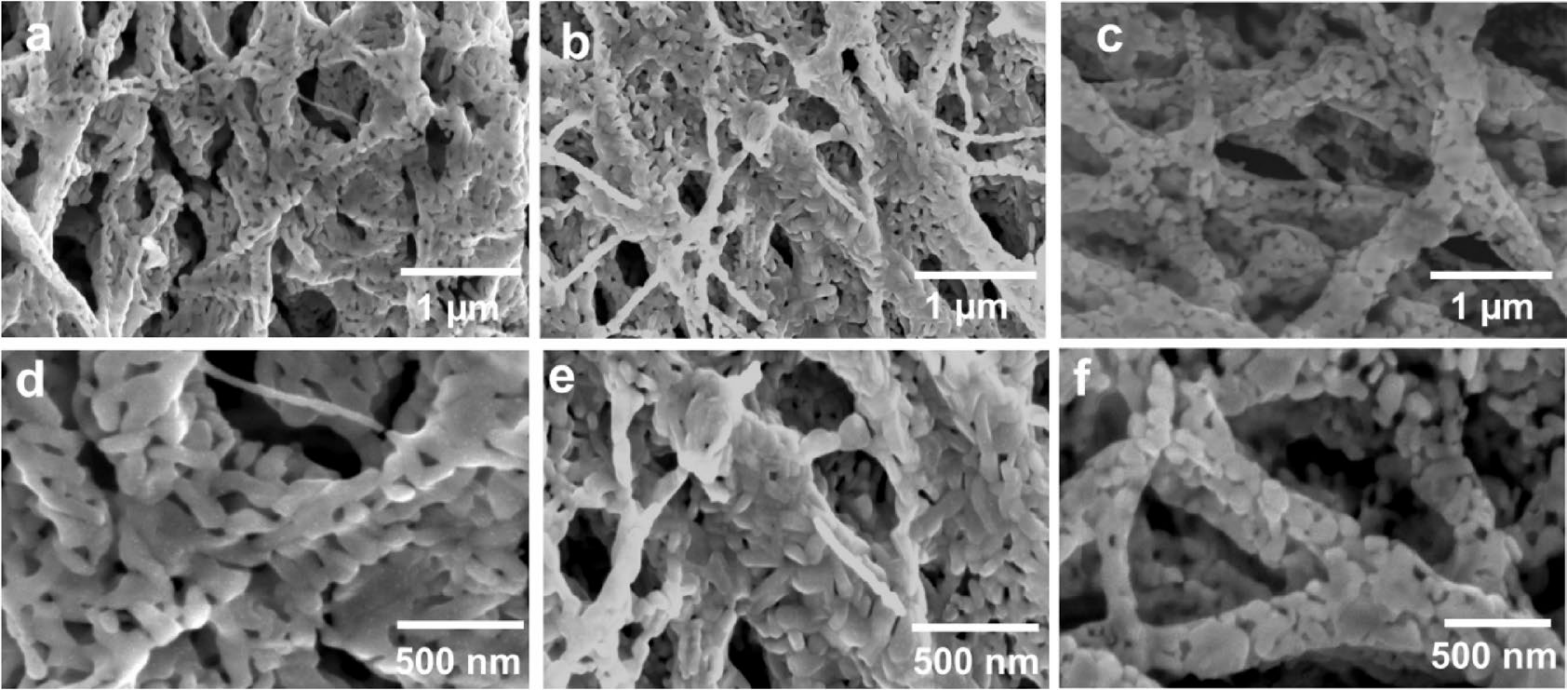
FESEM images of SFO nanofibers (a, d) NF21,(b, e) NF22,and (c, f) NF23.

Figure 4a-f illustrates the SEM images of fibers calcined at 800 ^o^C at the heating rates of 2 ^o^C/min (NF21), 6 ^o^C/min (NF22) and 10 ^o^C/min (NF23) at two different magnifications. The low magnification images (Fig. 4a, b,c) clearly show almost complete decomposition of PVP polymer and retention of the fiber structure even at a high heating rate with interconnected SFO rods and platelet morphology within the fibers. The average width of the fibers as calculated are 138 ± 7 nm, 152 ± 3 nm, and 242 ± 5 nm for NF21, NF22 and NF23, respectively and it increases with increase in heating rate. The PVP decomposition leads to the formation of porous fiber structure as observed and explained in the previous section. However, the SFO particles average size within the fiber decreases with increase in heating rates having values of 113 nm, 80 nm and 50 nm for NF21, NF22 and NF23, respectively as shown in Fig. 4d-f. This peculiar feature, where the width of the fiber increases and the mean size of the particles within the fiber decrease has been observed earlier by Jing et al.^9^. In their case the fiber width was varied as a function of PVP concentration, however in this case it is varied as a function of temperature and heating rate. PVP is a non-conducting polymer, which provides thermal insulation to the SFO precursors. The actual temperature seen by the precursor in PVP mixture depends on the heating rate. A slow heating rate appears to generate higher local temperature in the precursor leading to high porosity, smaller width and larger SFO particles compared to fast heating rate.

**Fig. 5:**
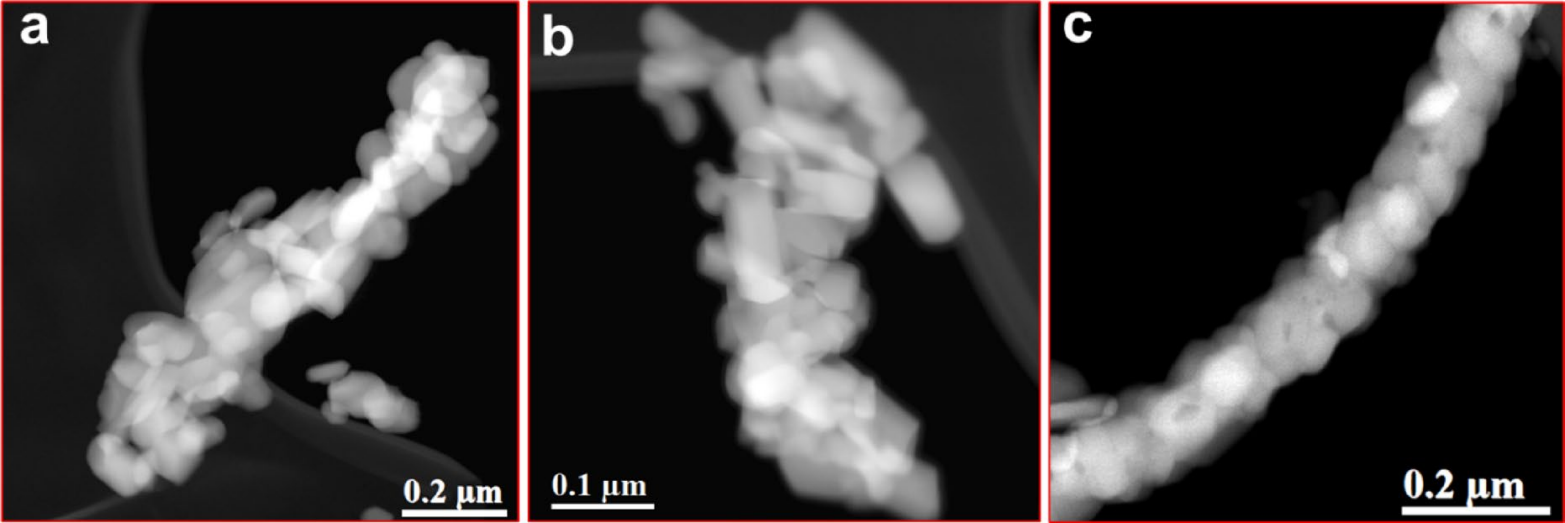
Scanning transmission electron microscopy (STEM) - High angle annular dark field (HAADF) imaging of the SFO nanofiber (a) NF21,(b) NF22,and (c) NF23.

Figure 5a-c shows the large-scale STEM-HAADF images of the nanofiber of SFO, NF21, NF22 and NF23. The uniform Z-contrast indicates the uniform distribution of the constituent nanoparticles. Particularly, NF23 shows the maximum particle uniformity (less porosity and low size distribution) compared to the other two. The low heating rate leads to higher local temperature, multiple nucleation centers of crystallites with non-uniform crystal growth and hence larger size distribution. This type of behavior has also been reported for iron oxide nanoparticles synthesized by thermal decomposition method, where nanoparticles have narrower size distribution at higher heating rate of 10 ^o^C/min compared to 1 ^o^C/min^27^. The free-standing nature of the nanofibers suggest the strong linkage of the constituent nanoparticle and robust nanofibers, which can maintain the structural integrity in the vacuum region of TEM.

**Fig. 6:**
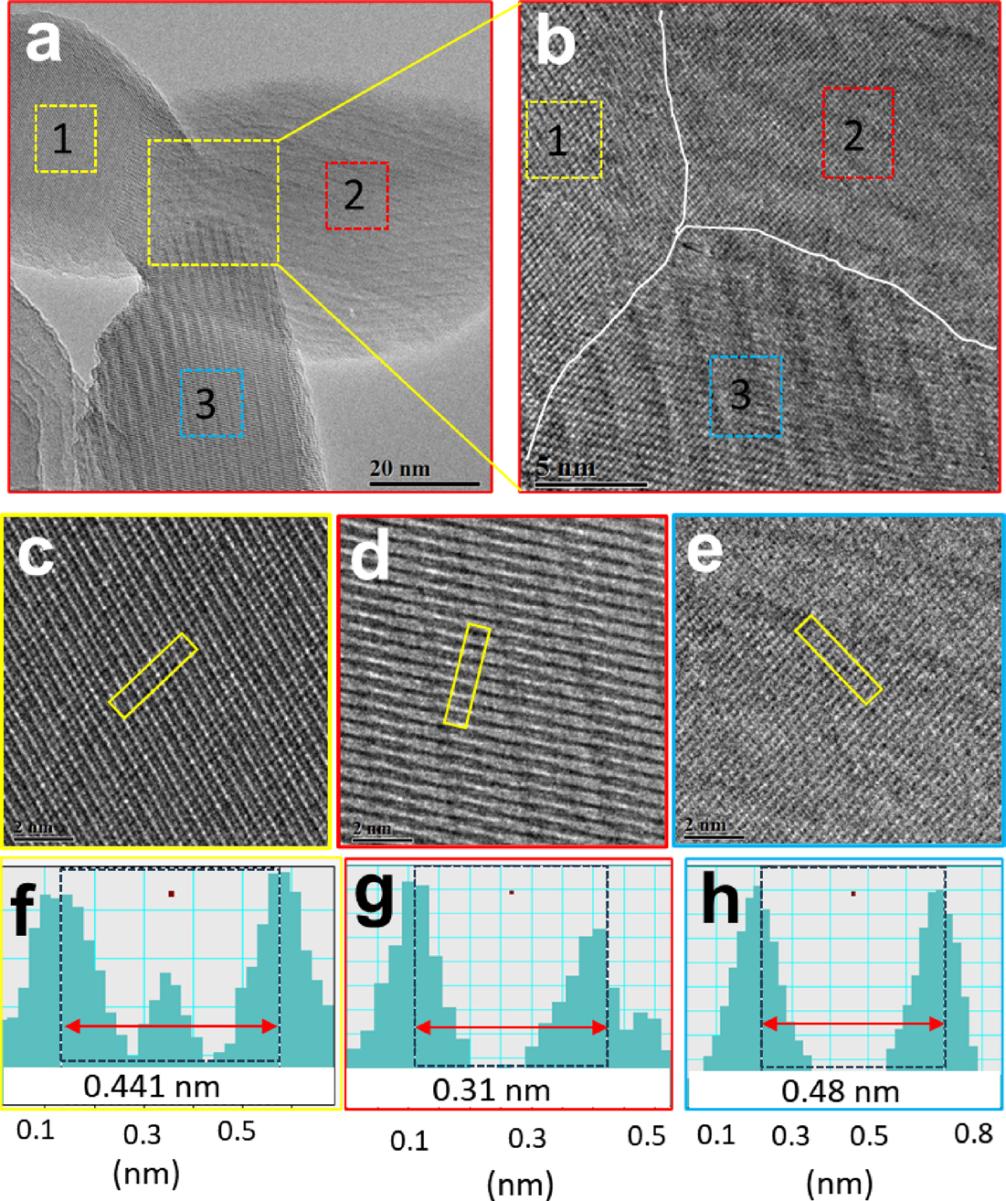
HRTEM image analysis of (a) tri-junction formation of nanoparticle in the NF22 nanofiber of SFO, (b) enlarged section of the junction of (a), (c-e) differential crystal orientations representative area 1–3, (f-h) measuring interplanar distance.

Figure 6a shows the HRTEM of NF22 SFO nanofiber, which reveals an interesting morphology where three different nanoparticles (represented as area 1–3 in a box) linked with each other in distinct crystal orientation form a trijunction. This is a unique observation and offers an insight to link the altered magnetic properties with morphology of the specimen. The phase contrast in HRTEM apparently indicates the distinct crystal orientation by showing well defined atomic orientations. Figure 6b shows the enlarged section of the junction, which appears to show merging of different single crystal orientation at the junction. The superimposed white line highlights the border area of each distinct orientation. Further analysis of the differential crystal orientations of representative area 1–3 are carried out using the enlarged section as represented in Fig. 6c-e. The crystal orientation are quantified by measuring the interplanar distance as depicted in Fig. 6f-h. The different interplane distance i.e. 0.436 nm, 0.31 nm, and 0.48 nm clearly indicates that the trijunction of the nanoparticles is formed by distinct crystal orientation.

**Fig. 7:**
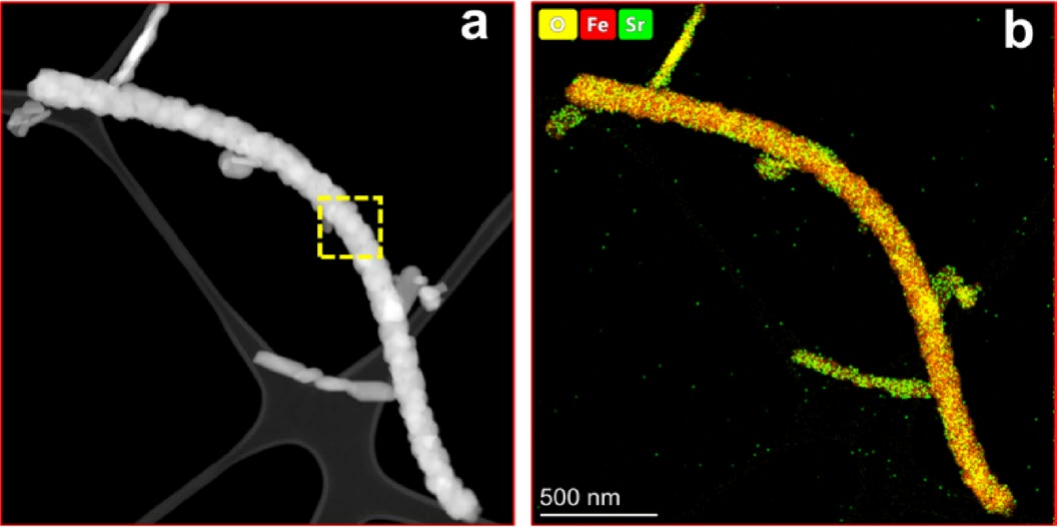
(a) STEM-HAADF image and (b) Compositional analysis of NF23 SFO nanofiber.

A detailed compositional analysis was carried out to understand the distribution of the constituent elements within the nanofiber of NF23. Figure 7a shows the high angle annular dark field image in scanning transmission electron microscope (STEM-HAADF) image of the NF23 sample. The STEM image shows a large thread of freestanding nanofiber with closely interlinked nanoparticles on the carbon grid. Figure 7b analyse the elemental distribution of the elements within the fiber, which reveals the uniform distribution of Sr, Fe, O across the nanofiber. The enlarged box section of Fig. 7a and EDX compositional analysis were shown in Supplementary information Fig. S1.

**Fig. 8:**
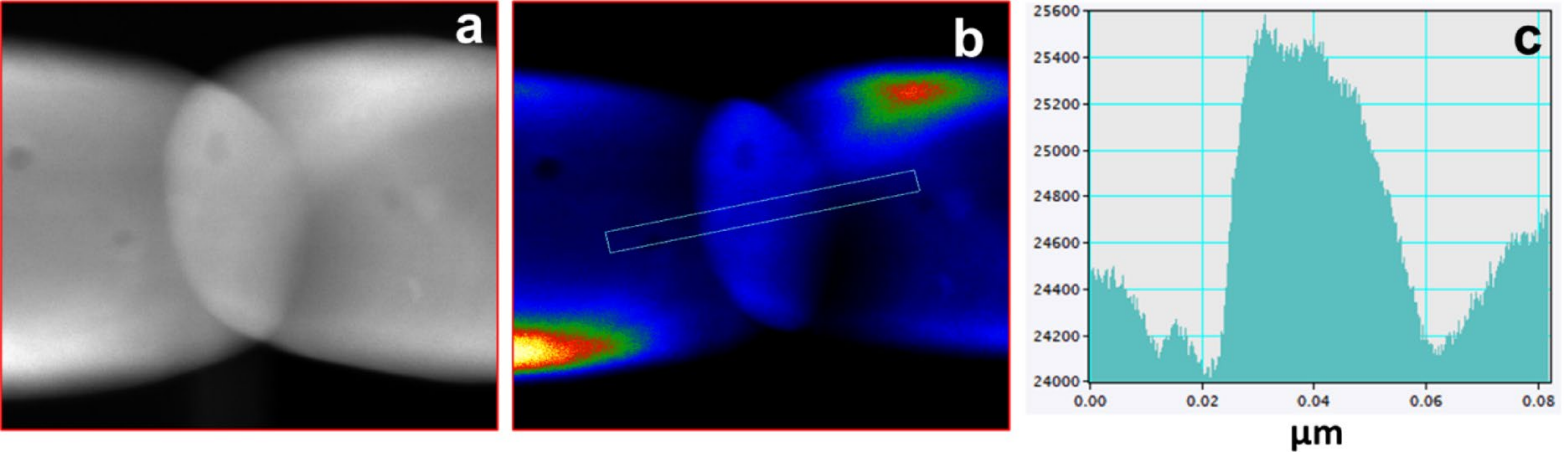
Imaging the interface of the nanoparticle in NF23 SFO nanofiber. (a) STEM-HAADF image of linked nanoparticles. (b) The contrast map showing the individual particle in the interface. (c) The thickness contrast map showing the presence of the nanoparticle at the interface.

The magnetic properties in such nanoparticle assembly get greatly affected by the interfaces between the nanoparticles. Therefore, a closer look is required at the interface of two neighboring nanoparticles. Figure 8a shows the HAADF-STEM image, which clearly reveals the nanoscopic morphology at the junction with a vivid linkage formation between the two neighboring nanoparticles. The STEM image shows a vivid linkage formation between two larger nanoparticles with an intermediate smaller nanoparticle of 40 nm along the fiber length having similar width. The contrast of image can be deceptive, as it appears the particles are overlapping. Therefore, to gain a deeper insight on the morphology at the interface, thickness contrast analysis was performed as shown in Fig. 8b with the line scan along the rectangular area shown in Fig. 8c. The contrast indicates that the thickness increases on the junction of the nanoparticles. The increased thickness contrast at the nanojunction suggests the presence of an additional particle, thus eliminating the possibility of the overlapping of the particle (Fig. 8a). It suggests that the interface is constituted of three different nanoparticles, where the small nanoparticle of size 40 nm offers a platform for the linkage of two bigger nanoparticles. The compositional variation due to the linkage of the nanoparticles is shown in Supplementary information Fig. S2.

### Thermal Characterization

As suggested from the analysis so far, PVP decomposition plays a crucial role in tuning the structure and morphology at the nanoscale. In order to get better insight into the PVP decomposition process and SFO phase formation, combined TG-DTA measurements were carried out on the as-spun fibers as shown in Supplementary Fig. S3. This illustrates the decomposition of PVP around 233 ºC – 344 ºC and phase formation of SFO at 422 ºC.

## Magnetic characterization

### Effect of calcination temperature.

**Fig. 9:**
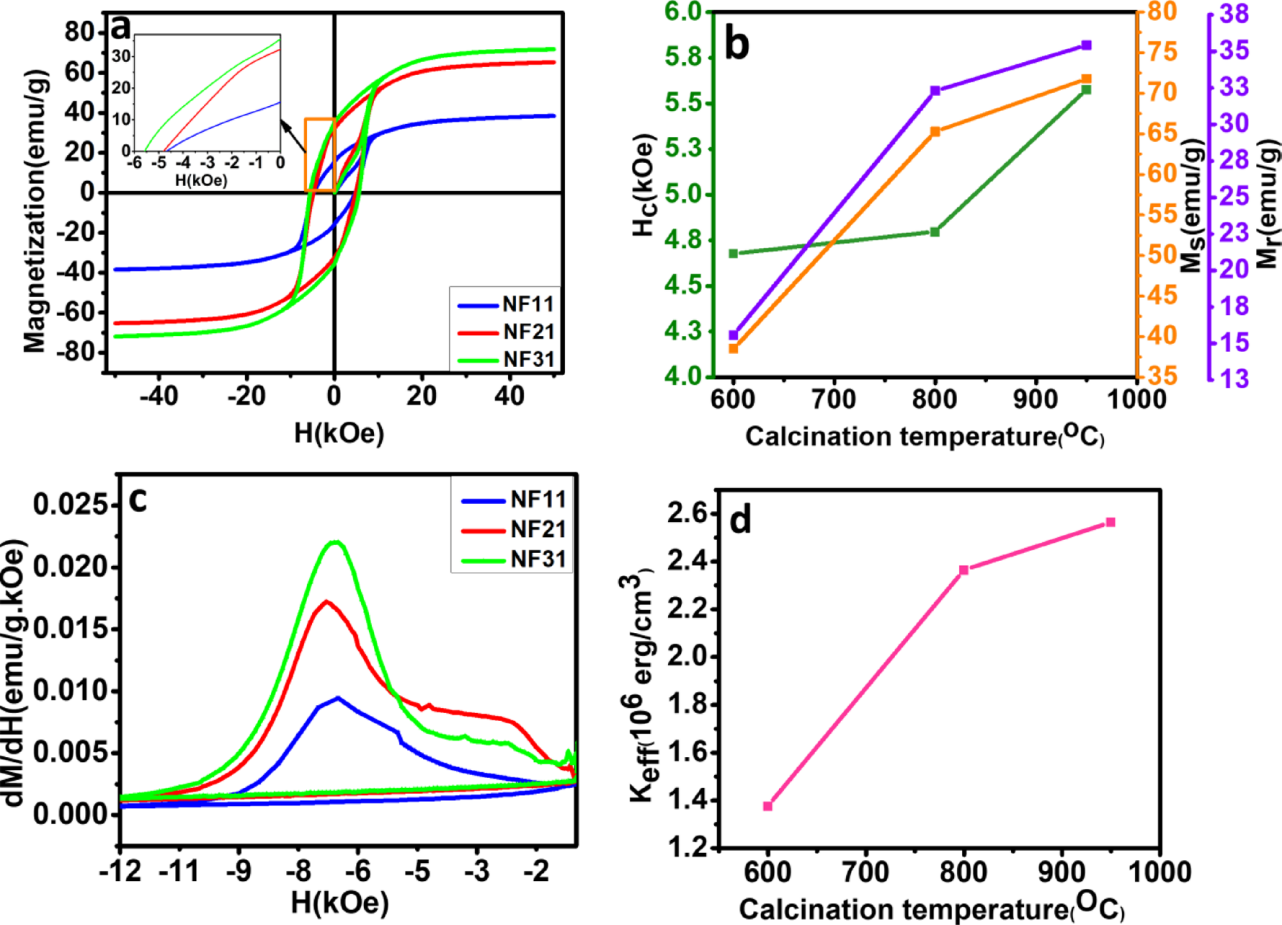
**Magnetization results** (a) Room temperature magnetic hysteresis (M-H loops) (b) Variation of magnetic properties (M_s_, M_r_, and H_c_) with calcination temperature (c) dM/dH of negative branch of M-H loop, and (d) variation of K_eff_ with calcination temperature of SFO nanofibers NF11, NF21, and NF31.

The room temperature magnetic hysteresis M-H loops of SFO nanofibers NF11, NF21, and NF31 are shown in Fig. 9a from which basic magnetic parameters, viz. saturation magnetization (M_s_), remanence (M_r_) and coercivity (H_c_) are extracted. All these parameters are mentioned in Table 3. Here, it should be emphasized that the magnetic properties and parameters obtained, correspond to the average magnetic response of the nanofiber ensemble and not individual nanofibers. Figure 9b shows the evolution of the parameters (M_s_, M_r_, M_r_/M_s_ and H_c_) for three calcination temperatures. The M_s_ and M_r_ values increase with increase in calcination temperature confirming the structural purity and decrease of secondary hematite phase at 950 ^o^C compared to 600 ^o^C as confirmed by XRD measurements. In fact the M_s_ value of 71.3 emu·g^− 1^ in NF31 is close to the theoretical value of 74.3 emu·g^− 1^ as per Stoner-Wohlfarth model and one of the highest among SFO nanoparticles prepared by chemical methods reported so far in the literature^19,28–30^. The high M_s_  value also suggests high degree of magnetic orientation and less defects in the crystal unit cells achieved through the simple heat treatment in presence of polymer. Moreover, the sample NF31 has the best magnetic parameters among the three due to better crystallinity and minute presence of hematite in it. The M_r_/M_s_ ratio for both NF21 and NF31 is close to 0.5, which indicates that the sample consists of randomly oriented domains as confirmed from FESEM and TEM images with randomly oriented nanofibers and nanoparticles within the fibers. The random orientation results in a distribution of the effective anisotropy of the ensemble.

Coercivity and the reversal mechanism is an important aspect for applications. The coercivity of the nanofibers depends on the intrinsic properties like magnetocrystalline anisotropy as well as the extrinsic properties such as size, shape, porosity and inter-particle magnetostatic interactions. The calcination temperature and heating rate modifies both the intrinsic and extrinsic properties, thus leading to a complex magnetization reversal process of the nanofiber ensemble^7,9,12,31^. Among these parameters, magnetocrystalline anisotropy is the dominant factor, which determines the coercivity. Although at nanoscale extrinsic properties might contribute significantly to the coercivity. NF11 calcined at 600 ^o^C has relatively higher H_c_ of 4.68 kOe due to the strong uniaxial anisotropy of SFO phase present, inspite it being the minor phase. This signals the dominant contribution of anistropy to the coercivity. The enhanced coercivity of NF21 and NF31 compared to NF11 can be attributed to the enhanced uniaxial anisotropy due the hexagonal phase formation as seen in XRD analysis. The size of the nanoparticles within the nanofibers for all the three samples are below the critical single domain size of SFO, which has a value between 500 nm and 1 μm as per literature^32^. That means the reversal process is expected to follow Stoner-Wholfarth model for single domain particles. However, the coercivity is still below the theoretical value of 7.4 kOe expected from the model, possibly due to the polycrystalline nature of the ensemble. As XRD analysis suggests, there should not be any variation of H_c_ between NF21 and NF31 as both have hexagonal crystal structure with little variation in the unit cell dimensions, where the uniaxial anisotropy field contribution is identical to the coercivity. However, an enhancement of coercivity of NF31 (5.57 kOe) compared to NF21 (4.79 kOe) is observed. Here, the additional role of morphology and the arrangement of nanoparticles within the fiber are responsible for the enhancement. NF31 has larger particle size (113 nm), narrower width of the fiber, uniform particle size and less porosity compared to NF21, which increases the effective anisotropy of the ensemble compared to NF21.

To investigate the variation in the coercive field in detail, the variation of first derivative of magnetization with magnetic field is plotted in Fig. 9c for the negative branch of the hysteresis loop. It shows two distinct regions with plateau in the low field region and a sharper peak in the high field region. The width of the peak represents the switching field distribution of the nanofiber assembly at different calcination temperatures, with the peak position assigned to the average switching field of the nanoparticles. It is clear that low calcination temperature NF11 and NF21 have broader switching field, which could be due to structural and morphological inhomogeneity in the sample and also contributes to the plateau observed in the low field region. NF31 shows a narrower width compared to other samples indicating that the uniformity and homogeneity is better, which corroborates with electron microscopy analysis as well. Further, effective anisotropy was calculated from Eq. (5), where the switching field was obtained from the peak position observed in Fig. 9c. The value of K_eff_ increases with calcination temperature as shown in Fig. 9d with the maximum value of 2.5 × 10^6^ erg.cm^− 3^ (close to the theoretical value of 3.5 × 10^6^erg.cm^− 3^) for NF31, which results into high H_c_ for NF31 compared to NF11 and NF21.

5 $$\:{H}_{SW}=\left(\frac{{K}_{eff}}{{M}_{S}}\right)$$.

**Table 3:** Magnetic properties (M_s_, M_r_, M_r_/M_s_ and H_c_) of the SFO nanofibers NF11, NF21 and NF31.

Sample names	M_s_(emu/g)	M_*r*_(emu/g)	M_*r*_/M_s_	H_c_(kOe)
**NF11**	38.49	15.58	0.40	4.68
**NF21**	65.26	32.29	0.49	4.79
**NF31**	71.79	35.42	0.49	5.57

In order to unravel the true magnetization reversal process and predicting the coercivity for NF21 and NF31 requires a multiscale physics approach due to wide variation in particle morphology, nanofiber alignment, cross linking between particles and complex inter-particle interaction. Here, a semi-empirical approach is followed to get better insight into the reversal process as described by Skomski et al.^33^. The reversal of magnetic nanostructures for single domain particles can be characterized by a length scale, namely coherent radius (*R*_*coh*_), which is related to fundamental length scale, the exchange length (*l*_*ex*_) and demagnetization factor, which is shape dependent as per the Eq. 6. For *R < R*_*coh*_, reversal takes place due to coherent rotation, whereas for *R > R*_*coh*_, curling mechanism dictates the reversal process.

6 $$\:{l}_{ex}=\sqrt{\frac{A}{{\mu\:}_{0}{M}_{s}^{2}}}$$.

$${R_{coh}}=3.665 \times {l_{ex}}{{ (for 1D nanostructure)}}$$

$${R_{coh}}=5.099 \times {l_{ex}}{{ (for spherical nanoparticles)}}$$

Here, *A* is the exchange stiffness (6 × 10^–12^ Jm^-1^) for SFO), *M*_*s*_ is volume saturation magnetization in Am^-1^ and *µ*_*0*_ is permeability of vacuum (4π×10^− 7^ NA^-2^). Based on Eq. (6), *l*_*ex*_ is of the order of 6.56 nm for NF21 and 5.96 nm for NF31 and the values of *R*_*coh*_ for 1D nanostructures are 23.97 nm and 21.784 nm, respectively, which are much lower than the particle size and fiber width. They satisfy the condition for *R > R*_*coh*_. Therefore, one can assume that despite being in single domain state the magnetization reversal follows the curling model rather than coherent rotation.

**Fig. 10:**
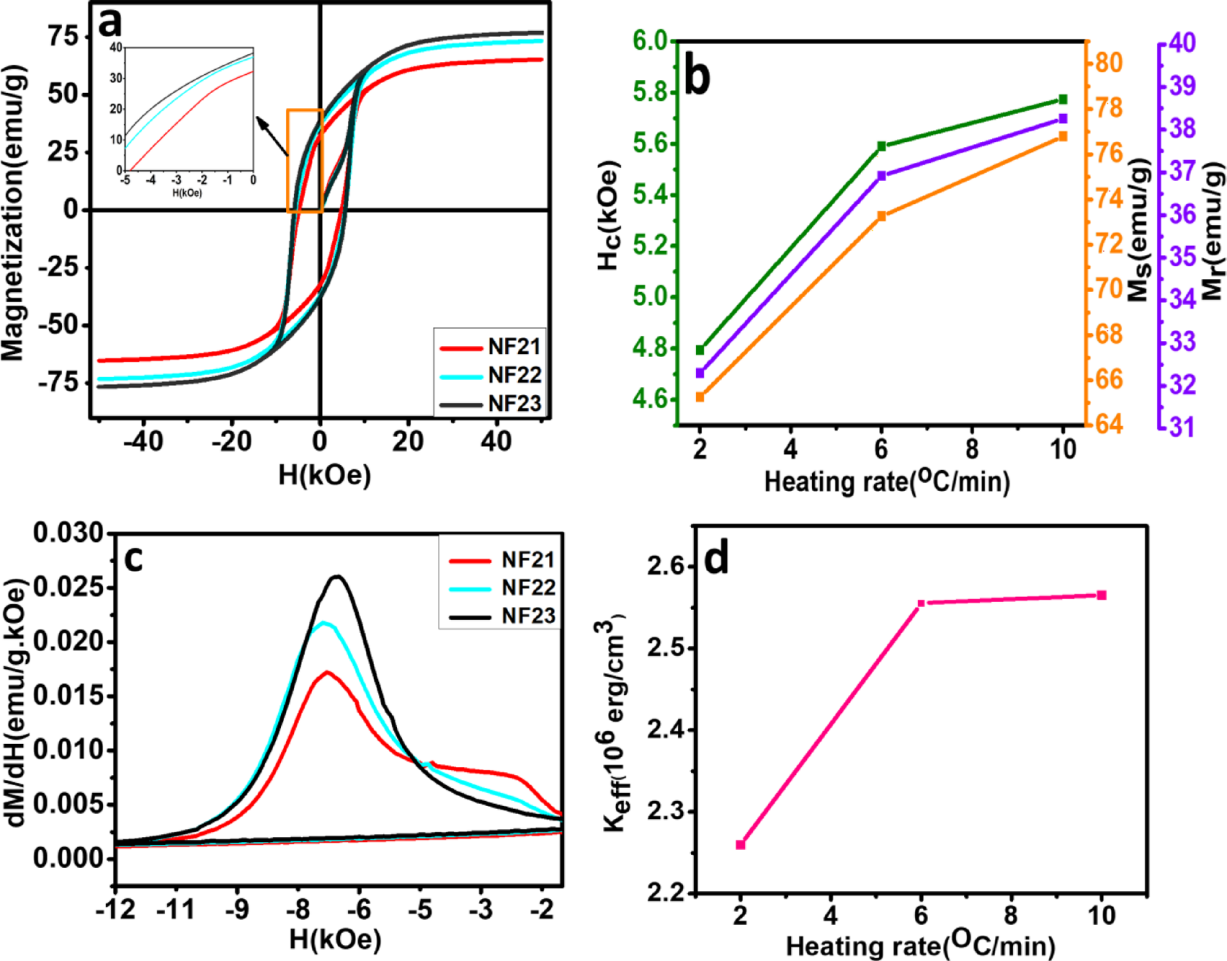
**Magnetization results** (a) Room temperature magnetic hysteresis M-H loops (b) Variation of magnetic properties (M_s_, M_r_, and H_c_) with heating rate (c) dM/dH of negative branch of M-H loop, and (d) variation of K_eff_ with heating rate of SFO nanofibers NF21, NF22, and NF23.

**Effect of heating rate.** The heating rate not only provides a simple yet effective way to tailor the structure, morphology and microstructure of the nanofibers as discussed earlier, but also significantly modify the magnetic properties. Figure 10a illustrates the room temperature hysteresis loop of the samples NF21, NF22 and NF23, where the calcination temperature was held fixed at 800 °C and the heating rate was varied from 2 °C/min, 6 °C/min and 10 °C/min, respectively. The magnetic parameters M_s_, M_r_ and H_c_ increases with increasing heating rate as depicted in Fig. 10b. All these parameters are mentioned in Table 4. The value of M_s_ increases from a modest 65.26 emu.g^-1^ to 76.79 emu.g^-1^ for NF23 (larger than NF31 sample), which is one of the highest experimental value reported for SFO nanosized particle assembly synthesized by chemical methods. This highlights the possibility to obtain better crystallinity and degree of alignment of nanoparticles within the fiber by varying the heating rate in presence of PVP at a lower calcination temperature of 800 °C compared to 950 °C. The M_r_/M_s_ ratio is still close to 0.5 for all the samples due to the random orientation of anisotropy axis of the ensemble.

The increase in H_c_ with heating rate is surprising having a maximum value of 5.77 kOe for NF23 and much higher than NF31 sample calcined at 950 °C as well. Typically, it is observed that H_c_ decreases with increase in M_s_ since the anisotropy field is inversely proportional to the saturation magnetization. However, in case of NF22 and NF23 both H_c_ and M_s_ increases. The high value of M_s_ indicates a high degree of crystal orientation in the material, which is achieved due to oriented anisotropic nanoparticles within the fibers. The orientation also leads to increase in the effective anisotropy enhancing H_c_. This enhancement is supported further due to low porosity of NF23 compared to other two samples, which minimizes the magnetic flux leakage and reduces the demagnetizing field. Thus, a combination of calcination temperature and heating rate is important to control the structure, morphology and the resultant magnetic properties of SFO.

To investigate the enhanced coercivity of the samples, the first derivative of the magnetization (dM/dH) was plotted versus magnetic field for the negative branch of the hysteresis loop as shown in Fig. 10c. NF23 has a sharper peak (narrow switching field distribution) compared to NF21 and NF22, which might be due to uniform particle size (narrow size distribution) and structural homogenity in the particles. NF21 has also low magnetocrystalline anisotropy, larger average particle size (113 nm) and high porosity in comparison to NF21 and NF23. For these two samples, the magnetocrystalline anisotropy increases along with reduction in particle size having values of 80 nm and 50 nm, respectively. This behavior shows that the particle size and magnetostatic interaction among them plays important role in determining the H_c_. It should be noted that FESEM analysis shows that NF23 has higher nanofiber width compared to NF22 and NF21. That means H_c_ is determined by the nanoparticle size rather than the width of the nanofibers. The *K*_*eff*_ was calculated using Eq. 5, where the switching field was obtained from Fig. 10c. The value of *K*_*eff*_ for the 3 samples are plotted in Fig. 10d with NF22 and NF23 having a larger value of 2.6 × 10^6^ erg.cm^-3^, much higher than all the nanofiber samples investigated in this work due to an optimized particle size and fiber width. The magnetization reversal process can be understood by calculating the *R*_*coh*_ using Eq. 6 for 1D nanostructure, which have values of 23.97 nm, 20.8 nm and 20.38 nm for NF21, NF22 and NF23, respectively; much smaller than the average particle sizes for all the samples. Therefore, the reversal is governed by curling rather than coherent rotation of magnetization as in the earlier samples.

Among all the investigated samples, NF23 nanofibers exhibit the most promising characteristics for permanent magnetic material: a high coercivity of H_C_ of 5.7 kOe, M_s_ = 76.79 emu·g^− 1^ and M_r_ = 38.26 emu·g^− 1^, which is obtained by tailoring the structure and morphology through simple heat treatment route.

**Table 4:** Magnetic properties (M_s_, M_r_, M_r_/M_s_ and H_c_) of the SFO nanofibers NF21, NF22, and NF23.

Sample names	M_s_( emu/g)	M_*r*_( emu/g)	M_*r*_/M_s_	H_c_( kOe)
**NF21**	65.26	32.29	0.49	4.79
**NF22**	73.26	36.92	0.50	5.59
**NF23**	76.79	38.26	0.49	5.77

## Experimental section

### Materials

Polyvinylpyrrolidone (PVP, Mw = 1,300,000, Alfa Aesar), strontium nitrate (Sr(NO_3_)_2_, ACS reagent, 99.0% purity, Alfa Aesar), iron(III) nitrate nonahydrate (Fe(NO_3_)_3_·9H_2_O, ACS reagent, 98.0% purity, Alfa Aesar) and N, N-Dimethylformamide (DMF, 99.0% purity, Alfa Aesar) were used as the initial precursors to obtain the as-spun nanofibers in this work as detailed here.

### Methods

**Fig. 11:**
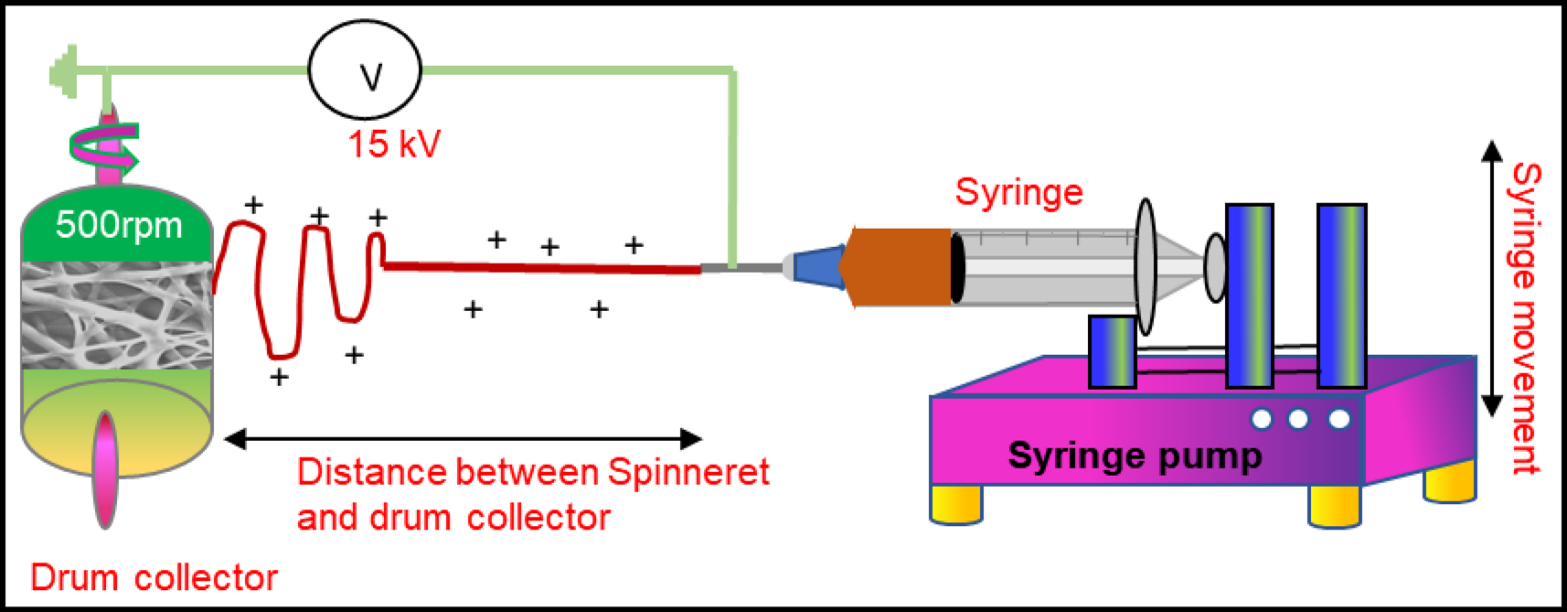
Schematic for synthesis process of SFO nanofibers by polymer-sol-assisted electrospinning technique.

The SFO nanofibers are synthesized by polymer-sol-assisted electrospinning technique followed by calcination. Figure 11 shows the schematic of the synthesis process of SFO nanofibers formation. A three-step process was followed to synthesize SFO nanofibers: preparation of precursor solution, production of as-spun nanofiber through electrospinning and preparation of SFO nanofibers by calcination of as-spun nanofibers.

**Step 1: Preparation of precursor solution.** To prepare the homogeneous solution, DMF is used as a solvent that efficiently dissolves both polymer carriers and inorganic precursors, hence ensuring uniform fiber morphology. A glass vial was filled with DMF solvent, followed by the addition of 0.1254 g of anhydrous strontium nitrate (Sr(NO₃)₂) and 2.874 g of iron(III) nitrate nonahydrate (Fe(NO₃)₃·9 H₂O) precursors (Sr to Fe ratio of 1:12). The resulting solution (solution A) was subjected to ultrasonication for 15 min. In a separate glass vial, DMF solvent and 15 wt% PVP polymer was added together and the mixture was stirred for 1 h at 75 °C (solution B). Solution A and B were mixed and stirred continuously overnight at 75 °C until a homogeneous viscous solution was achieved (solution C). This solution was stored in a syringe and fed into the electrospinning for the synthesis of nanofibers.

**Step 2: Production of nanofibers.** In order to obtain good quality nanofibers, it is necessary to optimize the electrospinning process parameters such as the applied voltage, flow rate, and distance between the needle and collector. Moreover, the viscosity and the conductivity of the solution could significantly affect the process as well^18^.Therefore a two-step optimization process was carried out as explained here. In the first optimization process nanofibers were produced using only solution B, i.e. of pure PVP nanofiber without the Sr and Fe nitrates. This step narrowed down the process parameters to an applied voltage window of 10–15 kV, needle to collector distance between 10 and 12 cm and a flow rate between 0.3 and 1 ml/hour^34,35^. In the second step, further optimization of solution C and process parameter were done to produce the nanofibers containing Fe and Sr. The optimized process parameters that resulted in continuous fibers without any defects (agglomeration) are 15 kV applied voltage, needle to collector distance of 12 cm and a flow rate of 1 ml/h. These process parameters were optimized for ambient conditions. The as-spun nanofibers obtained at this step are still non-magnetic in nature as there is no ferrite phase formation at this stage.

**Step 3: Preparation of SFO nanofibers.** To develop the final nanofibers with magnetic SFO phase, calcination steps were followed. The as-spun fibers were collected in alumina crucibles and calcined at 600 ^o^C, 800 ^o^C, and 950 ^o^C, where a slow heating rate was used to retain the nanofiber structure^8,9,34^.Therefore, a slow heating rate of 2 ^o^C/min was used in this case and these samples were labelled as NF11, NF21 and NF31, respectively. Another series of 3 samples were also prepared at a calcination temperature of 800 ^o^C with three different heating rates of 2 ^o^C/min, 6 ^o^C/min, and 10 ^o^C/min. These samples were labelled as NF21, NF22 and NF23, respectively. The heating rate controls the decomposition rate of the polymer and produces fibers with controlled porosity. All the heat treatment processes were carried in a muffle furnace for 1 h duration under normal atmospheric conditions. All the samples were allowed to cool down naturally without any forced cooling.

### Equipment

The structural characterization of synthesized SFO nanofibers was carried out by an x-ray diffractometer (XRD, Rigaku Miniflex) using Cu Kα radiation having a wavelength of 1.5406 Å in the 2θ range of 25^o^ to 70^o^ with the scan speed of 5 ^o^/min. The particle size and morphology were studied using field-emission scanning electron microscopy (FE-SEM, Thermoscientific) at an acceleration voltage of 10 kV. The mean particle size was calculated using ImageJ software by counting 50 particles for each composition from the micrographs^36,37^. The atomistic structural characterization and imaging of the sample is performed in an aberration correction transmission electron microscope, Cubed FEI-TITAN 60–300 kV. The HR-TEM and high angle annular dark field (HAADF) imaging is performed at 300 kV. A dedicated TEM specimen holder EI Super-X EDS, equipped with four-quadrant detector was utilized to improve the elemental mapping. The Gatan™ Digital Micrograph and Thermo Scientific™ Velox software suites were used for further post processing the TEM data sets^38^. The magnetic hysteresis (M-H) measurements were conducted using Magnetic Property Measurement System (MPMS) SQUID Dynacool under a magnetic field of ± 50 kOe at room temperature. The magnetic measurement of the nanofiber samples was carried out by taking few milligrams of powder sample into a 3 mm diameter VSM sample holder (Quantum Design), which was tightly pressed to ensure no mechanical movement of the powder in presence of magnetic field. This holder was positioned in a rod-shaped brass sample holder and securely clamped between two quartz rods tightly fitted within the holder. The thermal property of the as-spun fibers for reaction kinetics and crystallization process was recorded by thermogravimetric and differential thermal analysis TG-DTA (EXSTAR, SII 6300), with a precursor nanofiber placed in Al_2_O_3_ crucibles in the temperature range of 30 ^o^C – 1300 ^o^C at 10 ^o^C/min heating rate in air.

## Conclusions

Herein, unique way of tailoring the morphology of Strontium Hexaferrite, SrFe_12_O_19_ (SFO) nanofibers synthesized by the polymer-sol-assisted electrospinning technique followed by calcination were observed and characterized by XRD, FESEM, HRTEM, TG-DTA, and MPMS in detail. The calcination temperature and rate of heating has a significant role in tailoring the morphology of nanoparticles and nanofibers, by changing the shape of nanoparticles from irregular to rod-like or platelet shape. The nanofiber width reduced with increaseing calcination temperature, whereas it increased with variation in heating rate. On the other hand, avrerage size of the nanoparticles increased with increasing calcination temperature and decreased with variation in heating rate. The PVP decomposition played a vital role in making the nanofibers porous in structure with more porosity in case of slow heating rate at 2 ^o^C/min compared to heating rate at 10 ^o^C/min. STEM-HAADF images revealed the formation of linkages among nanoparticles within the fiber having uniform and stoichiometric distribution of Sr, Fe and O. HRTEM images provided a deeper insight into the formation of trijunction of nanoparticles with distinct atomic orientation. The XRD confirmed the phase formation of SFO phase with the impurity of hematite in it. The recorded M-H loop at room temperature for all the nanofibers showed the variation in magnetic properties due to the anisotropic morphology of SFO. The highest values of M_s_ and H_c_ were recorded for NF23 sample at the calcination temperature of 800 ^o^C and heating rate of 10 ^o^C/min (76.79 emu·g^− 1^, 5.77 kOe) due to the increase in the effective magnetic anisotropy and least secondary phase in it. The size of the SFO nanoparticles and its orientation within the fibers were the primary contributer to the simultaneous enhancement of the M_s_ and H_c_. Despite having nanoparticles below single domain size the reversal process is governed by curling mechanism rather than Stoner-Wohlfarth model due to the interlinked nanoparticle chain with 1-D geometry. This work leads to a unique pathway of synthesizing nanofibers with controlled and variable morphology that can tune the functional properties of a material at the nanoscale.

## Electronic supplementary material

Below is the link to the electronic supplementary material.

Supplementary Material 1

## Data Availability

The data supporting this article have been included as part of the Supplementary Information. It includes compositional analysis and EDX mapping of SFO nanofibers calcined at 800 °C with a heating rate of 10 °C/min, along with TG-DTA characterizations of as-spun nanofibers, providing deeper insights into the PVP decomposition process and the formation of the SFO phase.
