# Supplementary material for: Enhanced magnetic properties through tailoring of morphology of electrospun strontium hexaferrite nanofibers
Source: Sci Rep. 2025 Jul 2;15:23314. doi: 10.1038/s41598-025-04493-4 (PMC12223264; doi:10.1038/s41598-025-04493-4)
Supplement: Supplementary file 1 — Supplementary Material 1 [file 41598_2025_4493_MOESM1_ESM.docx]

**Supplementary Information**

**Enhanced magnetic properties through tailoring of Morphology of Electrospun Strontium Hexaferrite Nanofibers**

**Nishtha Vats, Saket S. Phadkule, Shrutidhara Sarma, Devendra S. Negi, Badri Vishal, Sampat R. Vadera^*^, Durgamadhab Mishra^*^**

^*^Department of Physics, Indian Institute of Technology, Jodhpur, India

This file contains Figures S1, S2, and S3 with legends.


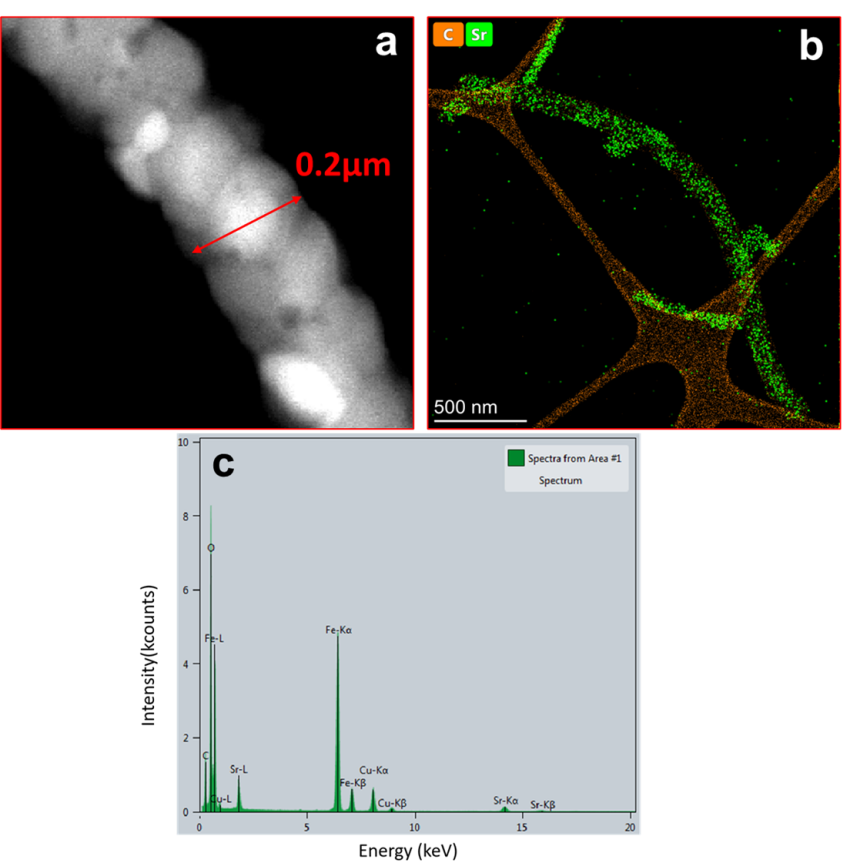


**Figure S1.** (a) The enlarge section of the STEM-HAADF image of NF23 nanofiber of Figure 7(a) (b) Compositional analysis (c) EDX compositional analysis of NF23 SFO nanofiber

Figure S1(a) shows the enlarge section of the high angle annular dark field image shown in main text of Figure 7(a) in scanning transmission electron microscope (STEM-HAADF) image of the NF23 sample. The average width of the nanofiber is ~ 0.2μm and matches with FESEM results. Figure S1(b) shows the background signal arising from the C grid and the dominating contrast due to the Sr distribution. The Sr atoms are uniformly distributed across the nanofiber. This suggests that the impurity hematite phase observed in XRD is low and difficult to find in the scanned nanofiber. Further, complete nanofiber scan was performed to access the total composition distribution. Figure S1(c) shows EDX intensity map (measured in the converged mode of the beam across the image shown in main text of Figure 7(a), where characteristics O-K, Fe-Kα and Sr-L edges were observed in the scanned energy range. The nanofiber exhibits a uniform distribution of constituent elements with substantial electron counts.


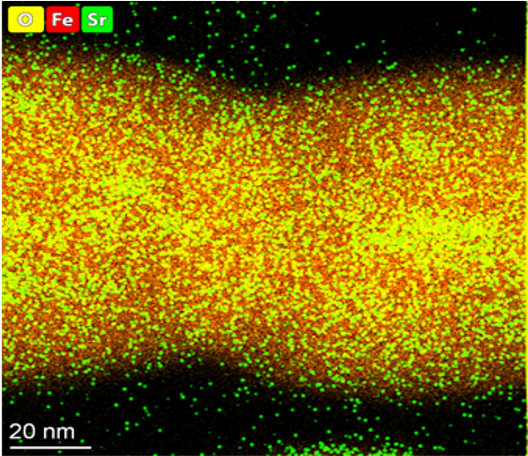


**Figure S2.** Elemental mapping of imaging the interface of the nanoparticle in NF23 SFO nanofiber.

The elemental analysis across the interface of STEM-HAADF image of linked nanoparticles (Figure 8(a)) is performed by the ED mapping and is shown in the Figure S2. The elemental mapping indicates a fairly homogeneous distribution of the constituent element. The Fe and O atoms are uniformly distributed. However, the Sr atoms shows some localized distribution in some pockets. Nevertheless, hematite phase was still difficult to ascertain at the interface.

**Thermal Characterization.** As suggested in the main text so far, PVP decomposition palys a crucial role in tuning the structure and morphology at the nanoscale. In order to get better insight into the PVP decomposition process and SFO phase formation, combined TG-DTA measurements were carried out on the as-spun fibers as shown in Figure S3.

Figure S3 illustrates the TG-DTA curves of the as-spun nanofibers, which reveals the temperature at which the SFO nanoparticle formation takes place and decomposition of the PVP is completed. TGA curve exhibits three steps of weight loss processes.  The first step of TG curve at 160°C shows a minor weight loss of 7.6%, which is caused by the evaporation of the DMF solvent^1^. The second step of weight loss (~37%) at 233ºC – 344ºC is due to the decomposition of PVP. In the DTA curve, the strong exothermic peaks at 303ºC and 422.1ºC are due to the decomposition of PVP and the transformation of the amorphous phase to crystalline phase and formation of SFO phase^1^. Above 422ºC, no more significant reduction in weight is observed, indicating that the entire decomposition of PVP and organic compounds has taken place, resulting probably in the formation of SFO and hematite. The hematite phase eventually converts to SFO phase at 800ºC or above.

**Figure S3.** TG-DTA curves of as-spun fibers

**Reference:**

1. Wang, Z., Zhao, L., Wang, P., Guo, L. & Yu, J. *J Alloys Compd* **687**, 2016, 541–547.
